# Supplementary material for: Resolving the full spectrum of human genome variation using Linked-Reads
Source: Genome Res. 2019 Apr;29(4):635–45. doi: 10.1101/gr.234443.118 (PMC6442396; doi:10.1101/gr.234443.118)
Supplement: Supplemental Material [file supp_gr.234443.118_Supplemental_longranger-2.2.zip › longranger-2.2.tar.bz2!/lr2.2-public/lib/stan/cmdstan/stan/doc/api/html/write__iteration__csv_8hpp_source.html]

Stan: src/stan/common/write\_iteration\_csv.hpp Source File


|  |  |
| --- | --- |
|  | Stan  2.5.0  probability, sampling & optimization |


- Main Page
- Related Pages
- Namespaces
- Classes
- Files

- File List
- File Members

All Classes Namespaces Files Functions Variables Typedefs Enumerations Enumerator Friends Macros Pages

- src
- stan
- common

write\_iteration\_csv.hpp

Go to the documentation of this file.

1 #ifndef STAN\_\_COMMON\_\_WRITE\_ITERATION\_CSV\_HPP

2 #define STAN\_\_COMMON\_\_WRITE\_ITERATION\_CSV\_HPP

3

4 #include <ostream>

5 #include <vector>

6

7 namespace stan {

8  namespace common {

9

10  void write\_iteration\_csv(std::ostream& output\_stream,

11  const double lp,

12  const std::vector<double>& model\_values) {

13  output\_stream << lp;

14  for (size\_t i = 0; i < model\_values.size(); ++i) {

15  output\_stream << "," << model\_values.at(i);

16  }

17  output\_stream << std::endl;

18  }

19  }

20 }

21

22 #endif

stan::common::write\_iteration\_csv

void write\_iteration\_csv(std::ostream &output\_stream, const double lp, const std::vector< double > &model\_values)

**Definition:** write\_iteration\_csv.hpp:10


---

[ Stan Home Page ]

*© 2011–2014,
Stan Development Team.*
